# Supplementary material for: Regulated membrane remodeling by Mic60 controls formation of mitochondrial crista junctions
Source: Nat Commun. 2017 May 31;8:15258. doi: 10.1038/ncomms15258 (PMC5460017; doi:10.1038/ncomms15258)
Supplement: Supplementary Information — Supplementary Figures and Supplementary Tables [file ncomms15258-s1.pdf]

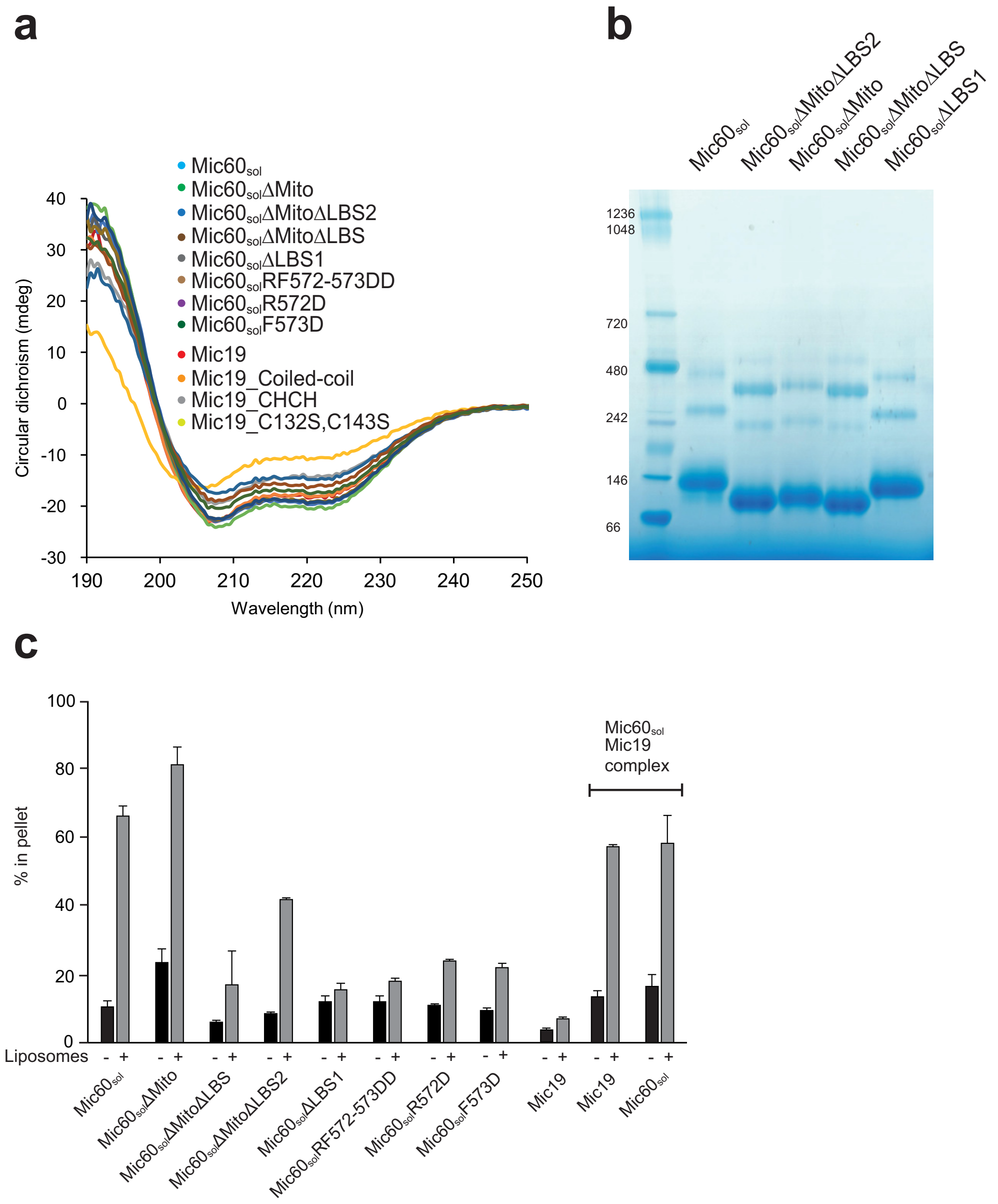

Supplementary Figure 1 - Assembly and membrane binding of Mic60$_{sol}$ and variants.

(**a**) CD measurements of the Mic60 and Mic19 constructs analyzed in this study indicated a mostly α-helical fold, as seen by the negative bands at 208 and 222 nm. Mutation of two cysteines in the CHCH domain of Mic19 led to a reduction in secondary structure, in agreement with a structural role of these two residues. (**b**) Blue native PAGE of Mic60 variants shows oligomerization independent of the mitofilin domain and the LBS. (**c**) Quantification of membrane binding from the liposome co-sedimentation assays shown in Fig. 1-3. The error bars denote the range of two independent measurements.

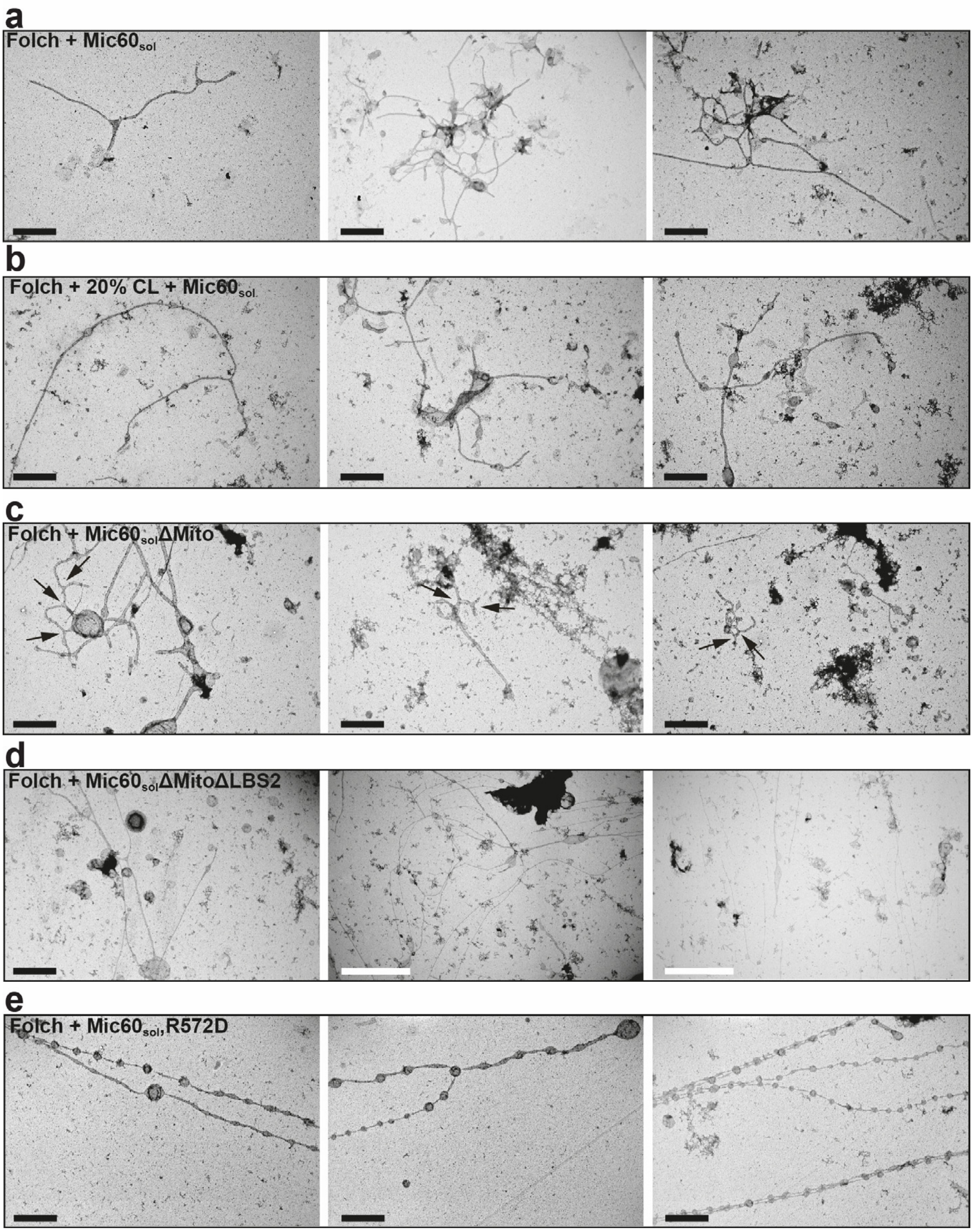
a
Folch + Mic60sol
b
Folch + 20% CL + Mic60sol
c
Folch + Mic60solΔMito
d
Folch + Mic60solΔMitoΔLBS2
e
Folch + Mic60sol,R572D

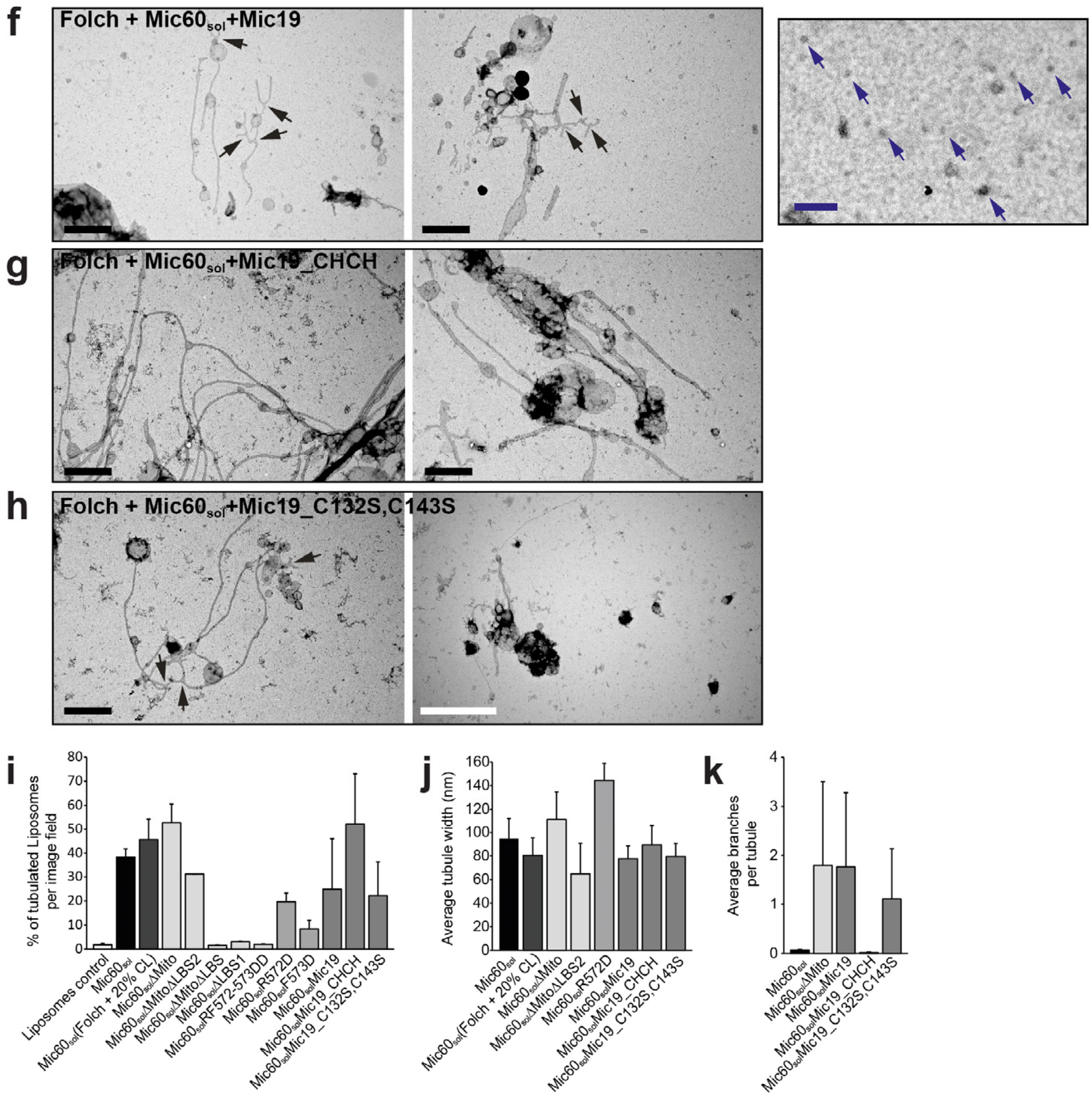

Supplementary Figure 2 - Mic60-mediated membrane remodeling.
(**a-h**) Representative electron micrographs of negatively stained liposomes after incubation with different Mic60 and Mic19 variants. Black arrows indicate detected branches and blue arrows fragmented liposomes (typical diameter 40-50 nm). Black scale bars represent 2 µm, white scale bars 10 µm and the blue scale bar represents 250 nm.(**i**) Quantification of the percentage of membrane tubules per image field for the indicated Mic60 variants. Only liposomes with a diameter ≥120 nm were considered in this analysis (tubulating Mic60 variants n~200, non-tubulating Mic60 variants n~1000, error bars denote the standard deviation). (**j**) Quantification of membrane tubule width for the indicated Mic60 variants (n=4 per tubule, with a total of 20 tubules per image field, error bars denote the standard deviation of the mean). For the R572D variant, only straight membrane tubules were quantified. (**k**) Quantification of branching points per tubule for the indicated Mic60 variants. n≥20, error bars denote the standard deviation.

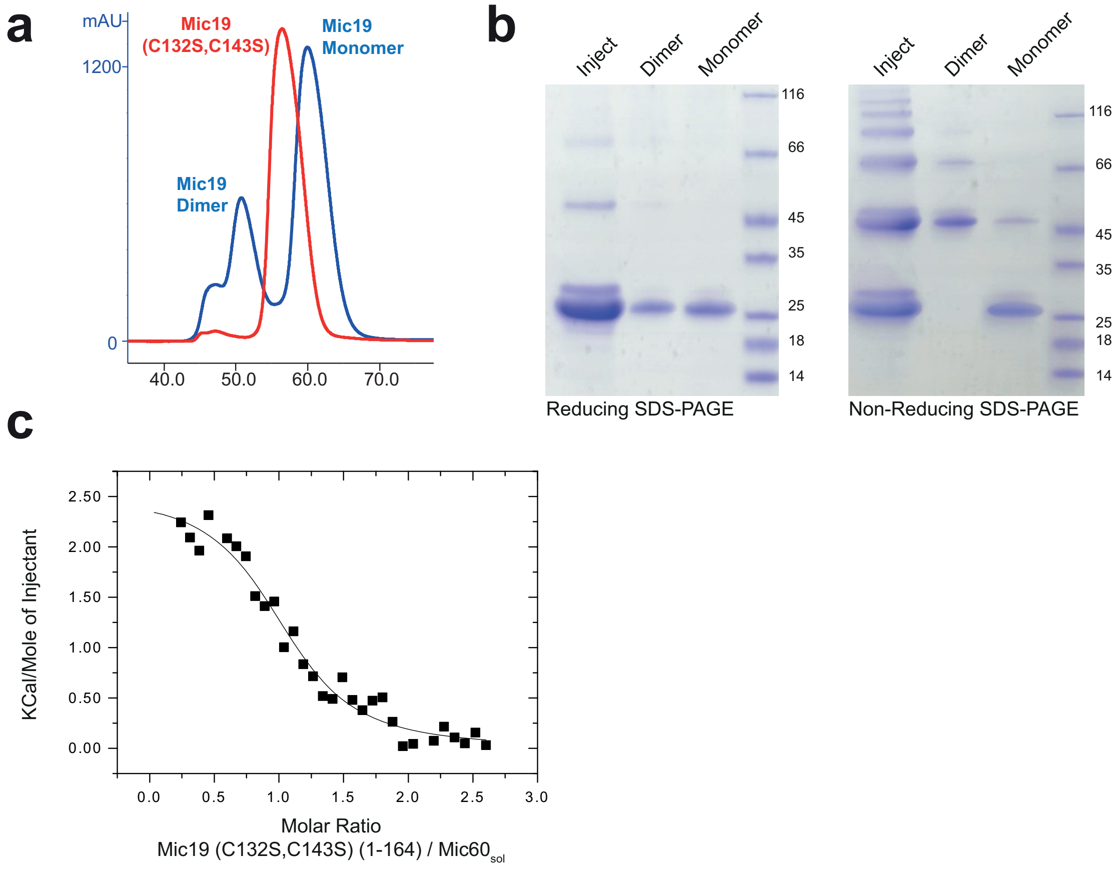

Supplementary Figure 3 - Characterization of Mic19 assembly.
(**a**) Gel filtration profile of Mic19 (blue) and the Mic19 C132S, C143S mutant (red). UV-absorption is indicated in milliabsorbance units (mAU) at the y-axis and the retention volume is plotted on the x-axis. Monomeric and dimeric peaks were detected for Mic19, whereas the Mic19 mutant formed only monomers. The slight shift of Mic19 C132S, C143S versus Mic19 monomer may indicate a partial unfolding of the CHCH domain in the mutated construct. (**b**) Reducing (left) and non-reducing (right) SDS-PAGE of the monomeric and dimeric Mic19 fractions after gel filtration. Inject refers to the starting material used for gelfiltration. (**c**) ITC experiments using $Mic60_{sol}$ and mutated Mic19 (C132S, C143S), as in Fig. 3b. The following values were obtained from the fitting: $K_D = 3.5\ \mu M \pm 0.8\ \mu M$, binding number n = 1.

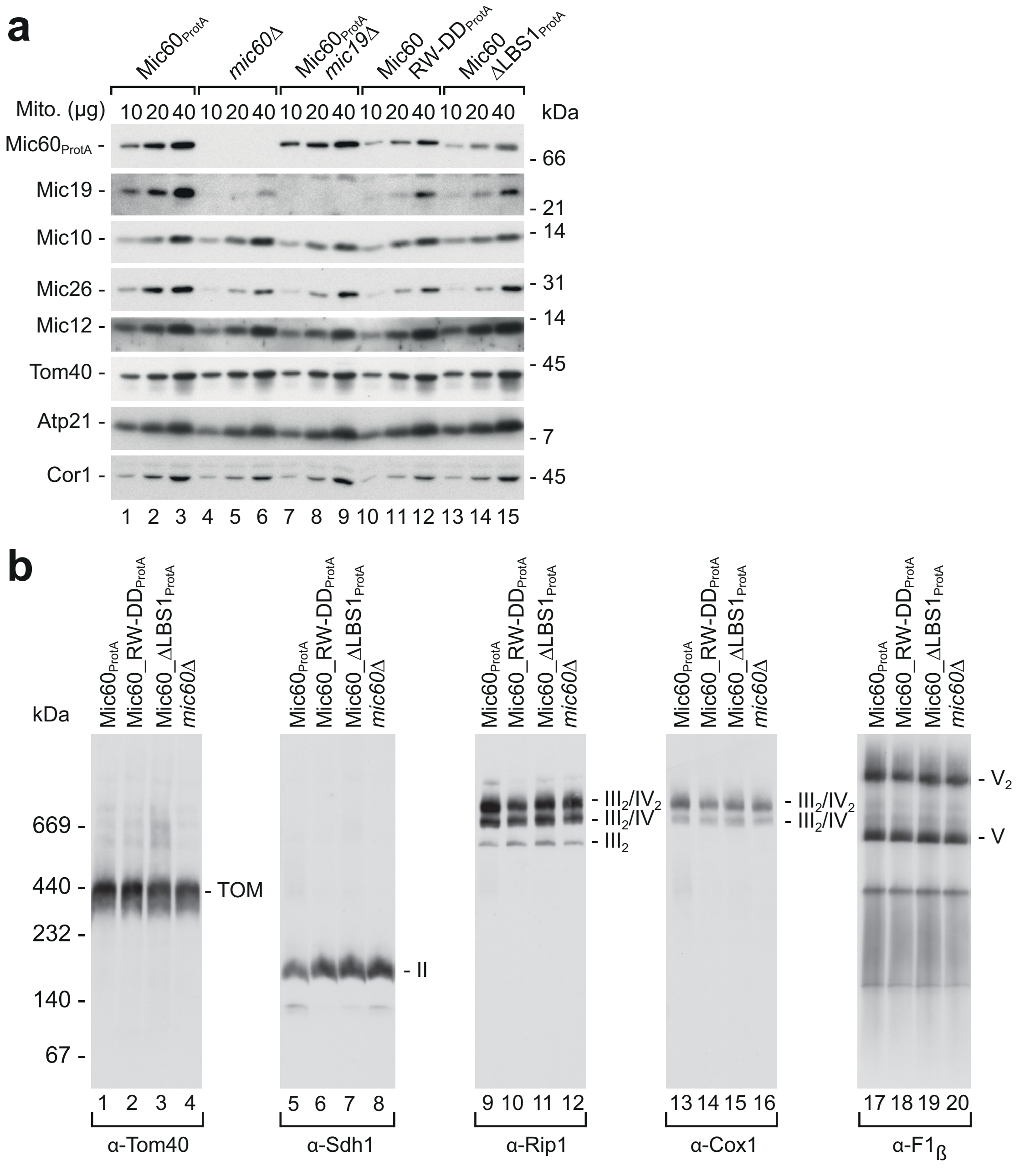

Supplementary Figure 4 - Expression and assembly analysis of MICOS. Mitochondrial steady state levels of the indicated individual proteins (**a**) or native protein complexes (**b**) were assessed by SDS-PAGE (**a**) or blue native-PAGE (**b**). $F_1\beta$ – β subunit of F_1F_o-ATP synthase / complex V. TOM – translocase of the outer membrane; II – complex II (succinate dehydrogenase); III_2 – complex III dimer (cytochrome bc_1 complex); III_2/IV – supercomplex consisting of a complex III dimer and one copy of complex IV (cytochrome *c* oxidase); III_2/IV_2 – supercomplex consisting of a complex III dimer and a complex IV dimer.

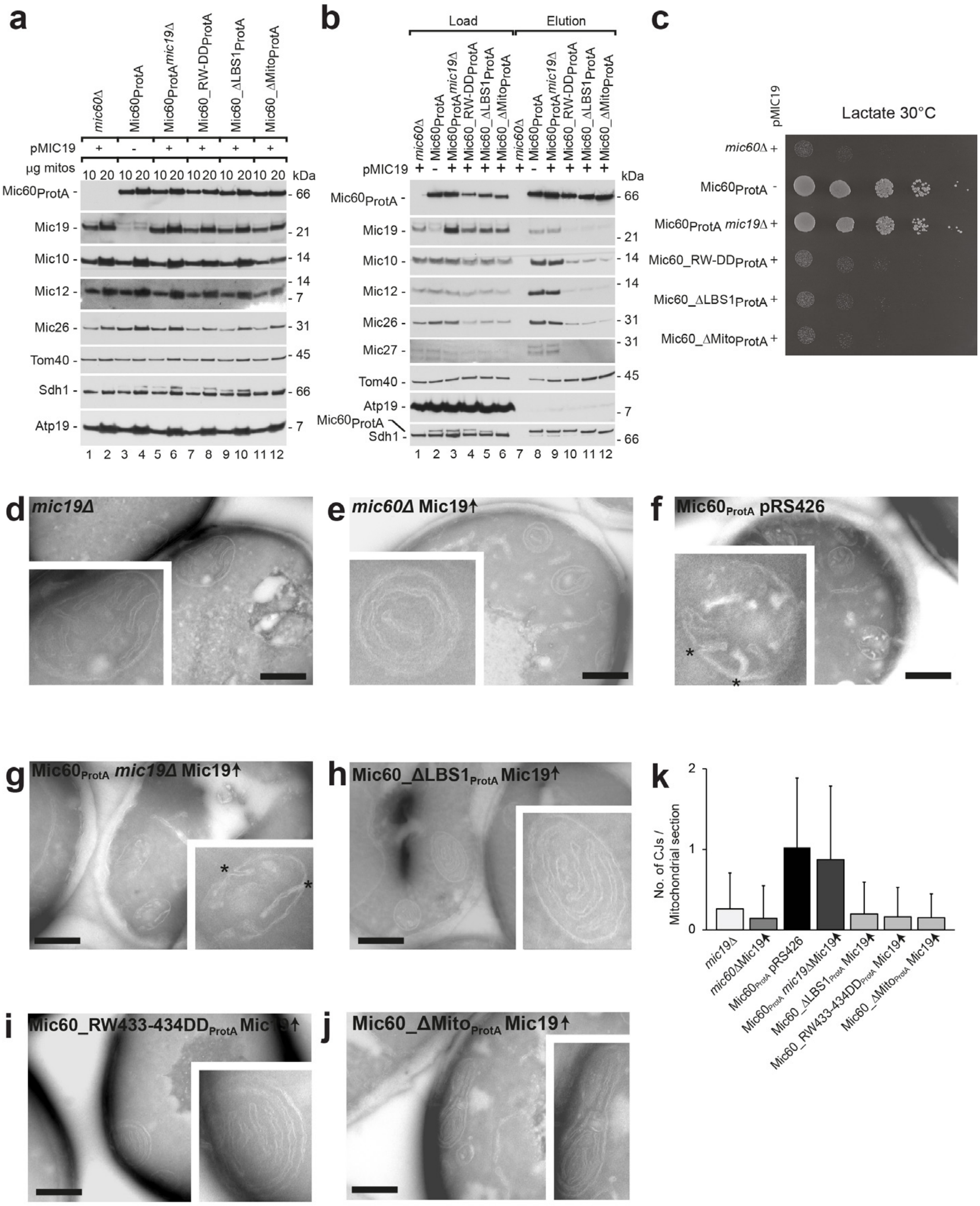

Supplementary Figure 5 – Re-expression of Mic19 in yeast strains with altered Mic60 proteins.

(a-c) Mic19 levels were restored in mutants with reduced endogenous Mic19 by Mic19 re-expression from a plasmid (pMIC19 +). Cells with normal endogenous Mic19 levels were supplemented with the empty vector (pMIC19 –). **(a)** Mitochondrial steady state protein levels of the indicated yeast strains, as described in Supplementary Fig. 4a. (**b**) MICOS integrity in the indicated yeast strains was assessed as described in Fig. 4a. Load 10%, eluate 100%. (**c**) Growth of the indicated yeast strains re-expressing Mic19 was assessed, as described in Fig. 4b. (**d-j**) Representative electron micrographs of mitochondria in ultrathin cryo-sections from yeast strains re-expressing Mic19, as described in Fig. 4c. Asterisks mark crista junctions. Scale bars represent 500 nm.

(**k**) Number of CJs in electron micrographs of mitochondrial sections from the various yeast strains as in Fig. 4g. n>=40, error bars indicate the standard deviation.

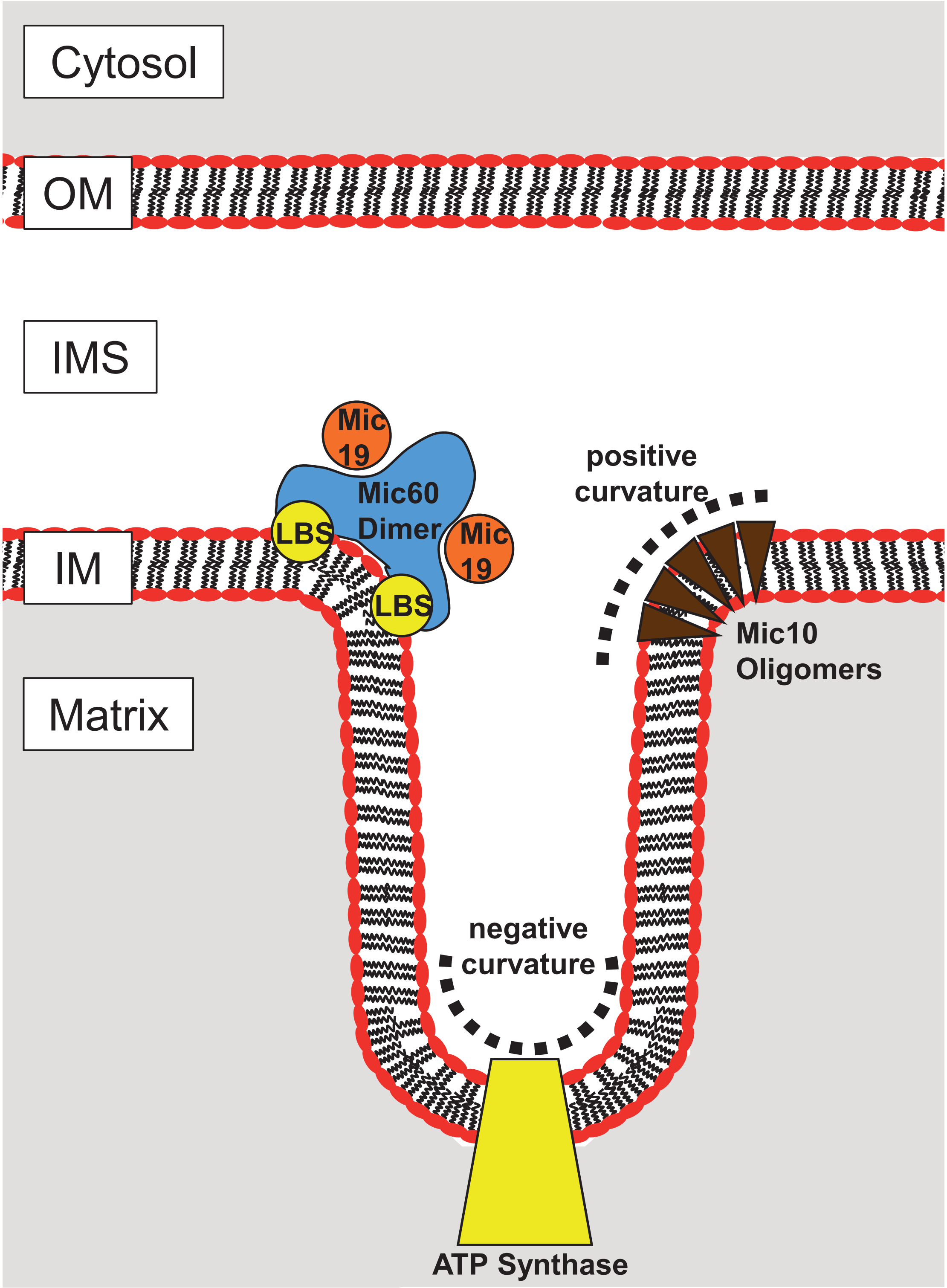

Supplementary Figure 6 – Stabilization of membrane curvature in mitochondrial cristae.
Cartoon illustration showing the suggested action of the Mic60-Mic19 complex in the generation or stabilization of membrane curvature at the rim of CJ. The cartoon also shows the location of ATP synthase dimers and the putative location of Mic10 oligomers which were also shown to contribute to membrane curvature stabilization.

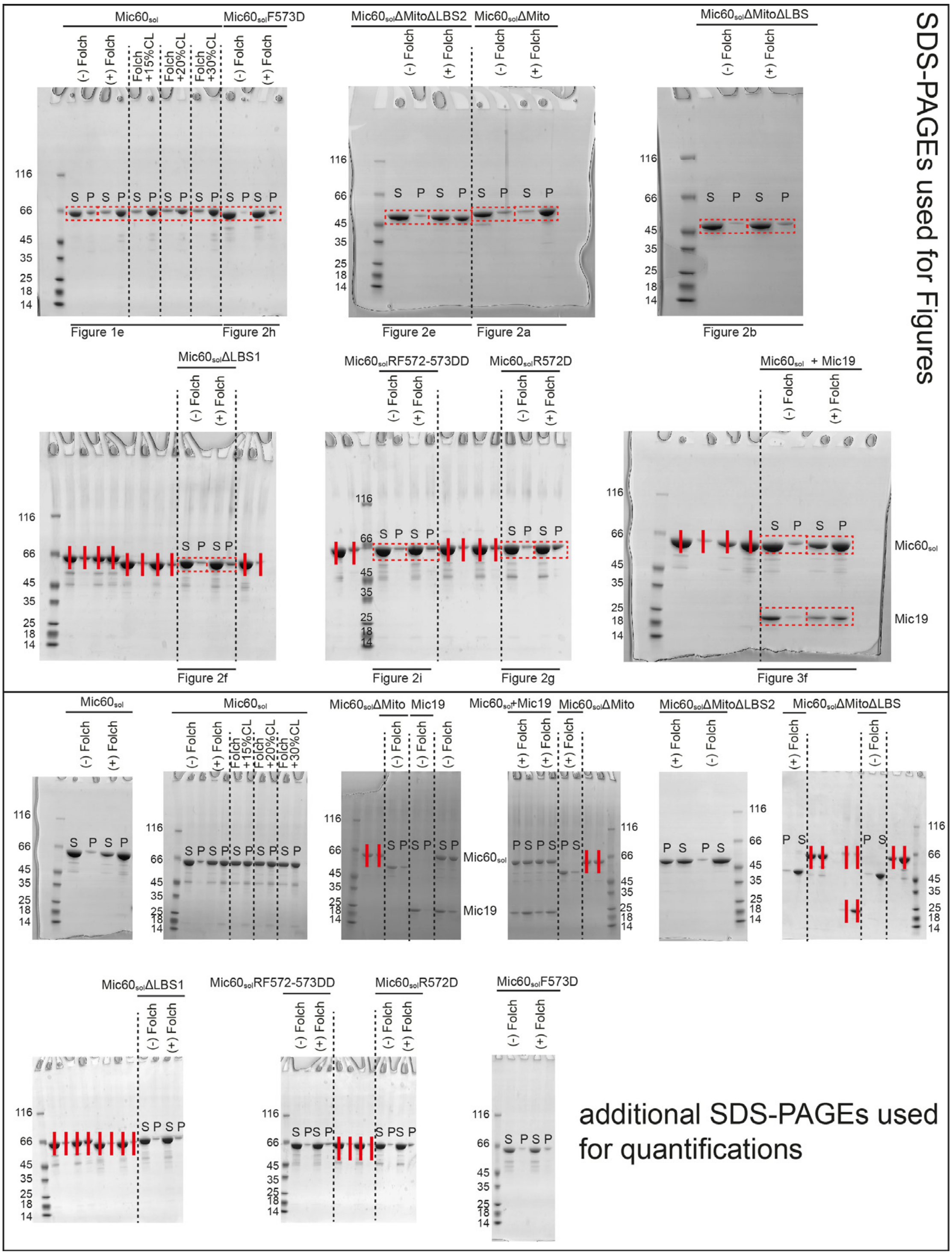
SDS-PAGEs used for Figures
Mic60$_{sol}$
Mic60$_{sol}$F573D
(-) Folch
(+) Folch
Folch +15%CL
Folch +20%CL
Folch +30%CL
Mic60$_{sol}$ΔMitoΔLBS2
Mic60$_{sol}$ΔMito
Mic60$_{sol}$ΔMitoΔLBS
116
66
45
35
25
18
14
S P
Figure 1e
Figure 2h
Figure 2e
Figure 2a
Figure 2b
Mic60$_{sol}$ΔLBS1
Mic60$_{sol}$RF572-573DD
Mic60$_{sol}$R572D
Mic60$_{sol}$ + Mic19
Mic19
Figure 2f
Figure 2i
Figure 2g
Figure 3f
Mic60$_{sol}$+Mic19
additional SDS-PAGEs used for quantifications

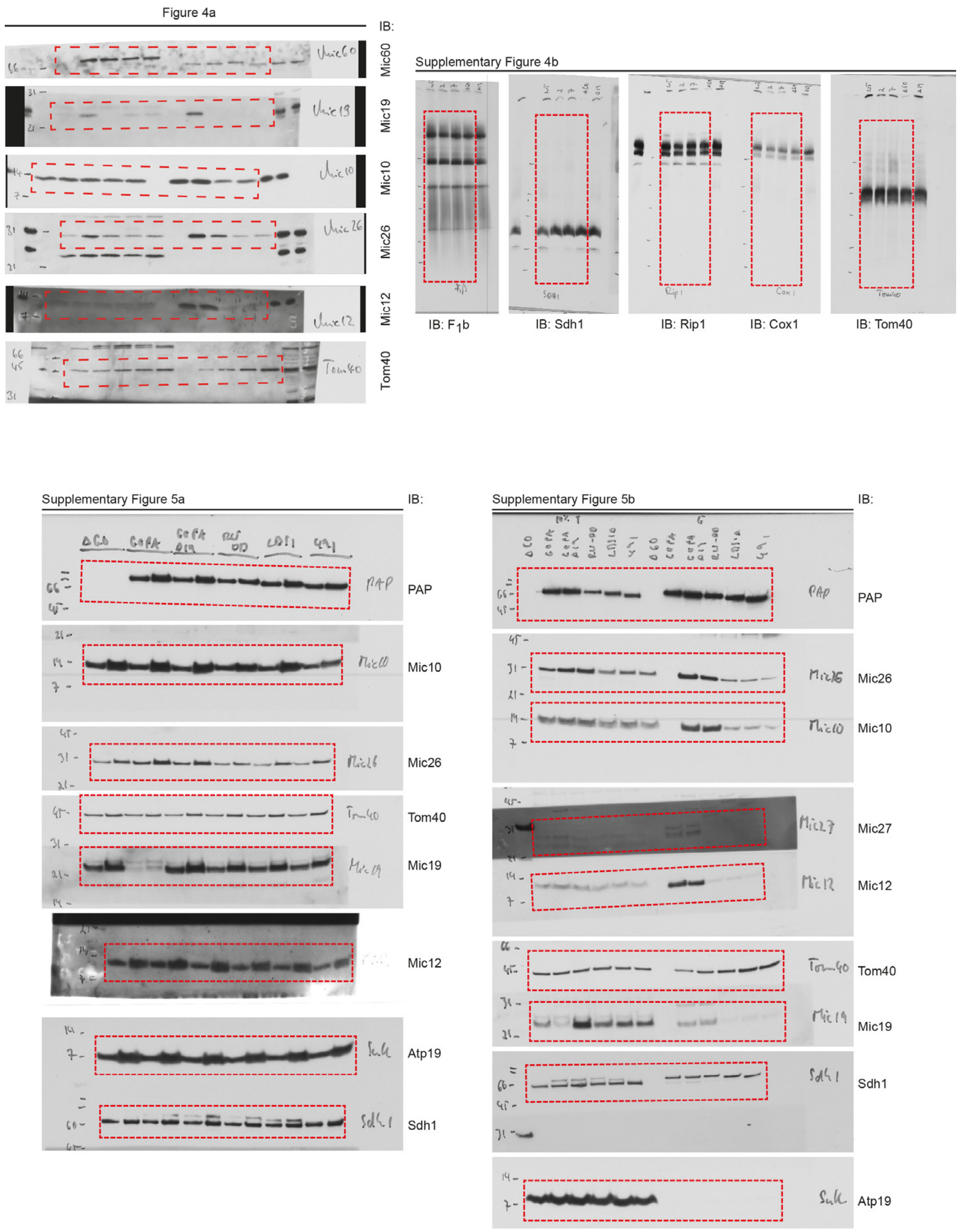

Supplementary Figure 7 – Original un-cropped SDS-PAGEs and Western blots. Red boxes refer to the cropped images shown in the indicated figures, red lines refer to lanes not used in the manuscript. S – supernatant, P – pellet. Molecular weight markers are indicated.

Supplementary Table 1 - Overview of liposome binding and deformation experiments.

Summary of liposome binding experiments using the co-sedimentation assay and liposome deformation experiments using negative stain electron microscopy.

	**Liposome binding**	**Liposome deformation**	**Deformation type**
**Mic60$_{sol}$**	Yes	Yes	Long tubules
**Mic60$_{sol}$ΔMito**	Yes	Yes	Tubules with branches
**Mic60$_{sol}$ΔMitoΔLBS**	No	No	
**Mic60$_{sol}$ΔLBS1**	No	No	
**Mic60$_{sol}$ΔMitoΔLBS2**	Yes	Yes	Long tubules
**Mic60$_{sol}$,R572D**	Reduced	Yes	Beads on a string
**Mic60$_{sol}$,F573D**	Reduced	partially	
**Mic60$_{sol}$,RF572-573DD**	Reduced	No	
**Mic19**	No	No	
**Mic60$_{sol}$ + Mic19**	Yes	Yes	Tubules with branches
**Mic60$_{sol}$ + Mic19 CHCH**	Yes	Yes	Long tubules
**Mic60$_{sol}$ + Mic19 (C132S/C143S)**	Yes	Yes	Tubules with branches

Supplementary Table 2 – Information and overview of used antibodies

Antigen	Dilution	Number/ Company	Secondary Antibody
Mic60	1:500	GR857-5	Rabbit
Mic10	1:200	GR3367-7	Rabbit
Mic12	1:250	GR3336-3	Rabbit
Mic19	1:250	GR3358-3	Rabbit
Mic26	1:250	GR3335-2	Rabbit
Mic27	1:400	GR3357-1	Rabbit
Tom40	1:1000	169-11	Rabbit
Sdh1	1:1000	GR1849-5	Rabbit
Cor1	1:400	GR371-6	Rabbit
Rip1	1:3000	GR542-3	Rabbit
Cox1	1:750	GR1538-2	Rabbit
Atp19	1:500	GR1961-3	Rabbit
Atp21	1:2000	138-9	Rabbit
$F_1\beta$	1:1000	GR861-3	Rabbit
PAP	1:200	Sigma P-1291	-
